# Costunolide reduces glycolysis-associated activation of hepatic stellate cells via inhibition of hexokinase-2

**DOI:** 10.1186/s11658-019-0179-4

**Published:** 2019-08-14

**Authors:** Dujing Ban, Shangbo Hua, Wen Zhang, Chao Shen, Xuehua Miao, Wensheng Liu

**Affiliations:** 1 Nanjing University of Chinese Medicine, Nanjing, 210023 China; 2 The Kunshan Hospital Affiliated to Nanjing University of Chinese Medicine, Kunshan, 215300 China; 3 The Nanjing Integrative Medicine Hospital Affiliated to Nanjing University of Chinese Medicine, Nanjing, 210014 China

**Keywords:** Hepatic fibrosis, Hepatic stellate cell, Costunolide, Glycolysis, Hexokinase 2

## Abstract

**Background:**

Hepatic stellate cell (HSC) activation is a central event during hepatic fibrosis. Aerobic glycolysis is one of its metabolic hallmarks. Blocking glycolysis is a novel therapeutic option for liver fibrosis. This study investigated the effects of costunolide, a natural product demonstrated to have hepatoprotective effects, on HSC activation and glycolysis.

**Methods:**

Primary HSCs were isolated from rats and cultured through 5 to 6 passages. Cell viability, activation markers, and glycolytic metabolism were examined in primary HSCs using various cellular and molecular approaches.

**Results:**

At 30 μM, costunolide reduced the viability of HSCs and inhibited the expression of α-smooth muscle actin and collagen I, two key markers of HSC activation. It also decreased glucose uptake and consumption, and reduced the intracellular levels of lactate in HSCs. At 10 mM, the glycolysis inhibitor 2-DG had a similar impact to costunolide at 30 μM: it significantly downregulated the expression of HSC activation markers. The combination of the two compounds produced more remarkable effects. Furthermore, costunolide repressed the expression and activity of hexokinase 2 (HK2), a pivotal rate-limiting enzyme that regulates glycolysis. However, overexpression of HK2 via plasmid transfection significantly reversed the costunolide-mediated downregulation of activation markers in HSCs, indicating that suppression of HK2 was required for costunolide to inhibit glycolysis-associated HSC activation.

**Conclusions:**

Our results show that costunolide can suppress HSC activation, and this is associated with inhibition of HK2, which blocks aerobic glycolysis. This suggests that costunolide is an antifibrotic candidate with potential for further development.

## Background

Hepatic fibrosis is a major contributor to the development of cirrhosis and liver cancer. It can be caused by continuous chronic liver injury and inflammation with various etiologies. During the pathological process, extracellular matrix (ECM) components, mainly type I and III collagens, are excessively produced and deposited in the liver, destroying its sinusoidal structure and function [1]. Hepatic stellate cell (HSC) activation has been defined as the most important event in liver fibrogenesis, because the original quiescent lipid droplet-rich HSCs proliferate and transdifferentiate to the pro-fibrogenic myofibroblasts, which are the primary source of ECM components and the pivotal players during fibrogenesis [2]. Attempts to elucidate the molecular mechanisms underlying HSC activation carry the hope of finding potential therapeutic targets for the management of liver fibrosis.

HSC activation is an energy-intensive process. Accumulating evidence suggests that activated HSCs use aerobic glycolysis as their major metabolic pathway in a phenomenon similar to the Warburg effect in cancer cells [3]. This metabolic switch is characterized by enhancement of glycolysis concomitant with repression of mitochondrial oxidative phosphorylation, even under normoxic conditions [4]. A number of intermediates of the glycolytic pathway are essential for the synthesis of amino acids, nucleotides and lipids, which are indispensible for maintaining cell functions [5]. The first rate-limiting step of glycolysis is catalyzed by hexokinase 2 (HK2), which effectively prevents glucose from leaving the cell, committing it to energy metabolism [6]. Thus, the highly proliferating HSCs can meet their energy and material requirements for cellular construction, despite the less efficient production of ATP in the glycolytic pathway. This suggests that inhibition of aerobic glycolysis could be a novel approach to reduce HSC activation and attenuate liver fibrosis [3].

Costunolide (C_15_H_20_O_2_) is a well-studied natural product that exhibits a broad range of biological activities, including anti-oxidant, anti-inflammatory and anti-tumor effects [7–11]. Interestingly, recent studies demonstrated that costunolide had potent hepatic protective effects. For example, it significantly reduced the serum levels of alanine aminotransferase and aspartate aminotransferase, and inhibited the hepatic expression of interleukin-1β and tumor necrosis factor-α in lipopolysaccharide- and d-galactosamine-induced acute liver injury [12]. Pretreatment with costunolide also inhibited hepatocyte apoptosis, which could be attributed to its anti-oxidative activity in this model [13]. However, the pharmacological utility of costunolide against liver disease and the underlying mechanisms are far from fully defined. Our study aimed to investigate the effects of costunolide on HSC activation with the hope of elucidating the mechanism of its anti-fibrotic potential.

## Methods

### Reagents and antibodies

Costunolide (purity > 98%) and the glycolysis inhibitor 2-deoxy-D-glucose (2-DG) were purchased from MedChemExpress. The two compounds were dissolved with dimethyl sulfoxide (DMSO) for the experiments. Treatment with DMSO alone was used as vehicle control. Rabbit polyclonal antibodies against α-smooth muscle actin (α-SMA, cat. no. 55135–1-AP), collagen I (cat. no. 14695–1-AP), HK2 (cat. no. 22029–1-AP) and glyceraldehyde phosphate dehydrogenase (GAPDH, Cat. No. 10494–1-AP), and secondary antibody HRP-conjugated Affinipure Goat Anti-Rabbit IgG (H + L) (cat. no. SA00001–2) were purchased from Proteintech Group.

### Culture of primary HSCs and cell transfection

Animal studies were performed in compliance with the ARRIVE guidelines and the Basel Declaration. Experimental procedures were approved by the Institutional and Local Committee on the Care and Use of Animals of Nanjing University of Chinese Medicine (ACU180905) on September 28, 2018. All animals received humane care according to the National Institutes of Health guidelines. Primary HSCs were isolated from male Sprague-Dawley rats according to the reported methods and procedures [14, 15]. The isolated HSCs were cultured in Dulbecco’s modified Eagle medium (DMEM; Invitrogen) with 10% fetal bovine serum (FBS) and 1% antibiotics. They were grown in a 5% CO_2_ humidified atmosphere at 37 °C. HSCs from passages 5 or 6 were deemed to be sufficiently activated, and were used for the experiments. The overexpression plasmid of HK2 pcDNA3.1(+)-HK2 was purchased from Obio Technology. Transfection with the overexpression plasmid of HK2 was conducted using Lipofectamine 2000 Transfection Reagent (Life Technologies) following the manufacturer’s instructions.

### Determination of cell viability

HSCs were treated with costunolide at 10, 20 and 30 μM for 24 h, and the cell viability was evaluated using MTT assays according to the reported methods [16]. Briefly, the medium of the treated HSCs was replaced with 100 μl phosphate-buffered saline (PBS) containing 0.5 mg/ml 3-(4,5-dimethylthiazol-2-yl)-2,5-diphenyl tetrazolium bromide (MTT; Sigma) and the cells were then incubated at 37 °C for 4 h. The crystals were dissolved with 200 μl dimethylsulfoxide. The spectrophotometric absorbance at 490 nm was measured using a SPECTRAmax microplate spectrophotometer (Molecular Devices). Cell viability was expressed as a percentage of the control.

### Measurement of glucose metabolism

HSCs were treated with costunolide at 10, 20 and 30 μM for 24 h. The uptake of glucose was measured using Abnova assay kits. Glucose consumption was measured using Shanghai Meilian Biology Technology ELISA kits for determining the intracellular activity of glucose oxidase (GOD). All experiments were performed following the manufacturers’ protocols.

### Measurement of intracellular lactate

HSCs were treated with costunolide at 10, 20 and 30 μM for 24 h. Lactate levels in lysates of the HSCs were measured using Nanjing Jiancheng Bioengineering Institute kits according to the manufacturer’s instructions.

### Measurement of intracellular HK2 activity

HSCs were treated with costunolide at 10, 20, and 30 μM for 24 h. The intracellular activity of HK2 was detected using the kits purchased from Shanghai Meilian Biology Technology following the protocols provided by the manufacturer. Briefly, 40 μl diluent and 10 μl cell lysate sample were added to the each well of an enzyme-labeling plate followed by a 30-min incubation at 37 °C. The liquid was discarded from each well, followed by five washes with 1x washing solution. Next, 50 μl HRP-conjugate reagent was added to each well, followed by a 30-min incubation at 37 °C. The next step was a further five washes with 1x washing solution. Then, 50 μl chromogen solution A and 50 μl chromogen solution B were added to each well followed by a 15-min incubation at 37 °C. 50 μl stop solution was added to dominate the reaction in a 15-min incubation at 37 °C. The spectrophotometric absorbance at 450 nm was measured using a Molecular Devices SPECTRAmax microplate spectrophotometer.

### Real-time PCR

HSCs were treated for 24 h with: 10, 20 or 30 μM costunolide; with 30 μM costunolide and/or 5 mM 2-DG; or with 30 μM costunolide and/or transfection with HK2 overexpression plasmid. Total RNA was prepared using Trizol reagent (Invitrogen), and first-strand cDNA was synthesized with 1 μg of total RNA using PrimeScript RT reagent kits (Takara Bio). Real-time PCR was conducted using the IQTM SYBR Green supermix (Quanta) and iQ5 detection system (Bio-Rad Laboratories). The reaction mixtures contained 7.5 μl SYBR Green I dye master mix, 2 pM forward primers, and 2 pM reverse primers. The thermocycling conditions included denaturation at 50 °C and 95 °C for 10 min, 40 cycles at 95 °C for 15 s, and 60 °C for 1 min. The relative levels of mRNA were determined using the 2^-ΔΔCT^ method with GAPDH as the invariant control. The primers (Sangon Biotechnology) were: α-SMA: (forward) 5′-CCGACCGAATGCAGAAGGA-3′, (reverse) 5′-ACAGAGTATTTGCGCTCCGGA-3′; collagen I: (forward) 5′-CCTCAAGGGCTCCAACGAG-3′, (reverse) 5′-TCAATCACTGTCTTGCCCCA-3′; HK2: (forward) 5′-CTGCCACAGCATGATGAGGATTGAT-3′, (reverse) 5′-GCCAGGATGGCTGAGATCACCAC-3′; and GAPDH: (forward) 5′-GGCCCCTCTGGAAAGCTGTG-3′, (reverse) 5′-CCGCCTGCTTCACCACCTTCT-3′. Each sample had five duplicates and experiments were performed in triplicate.

### Western blot

HSCs were treated for 24 h with: 10, 20 or 30 μM costunolide; with 30 μM costunolide and/or 5 mM 2-DG; or with 30 μM costunolide and/or transfection with HK2 overexpression plasmid. The whole-cell lysates were prepared using radioimmunoprecipitation analysis buffer containing protease inhibitors. BCA assay kits (Pierce) were used to measure the protein concentrations. Proteins (50 μg/well) were separated on SDS-polyacrylamide gel via electrophoresis followed by transferring the proteins to a PVDF membrane (Millipore). The membranes were blocked with 5% skim milk in TBS-T solution. The target proteins were monitored using the primary antibodies against α-SMA (dilution 1:1000), collagen I (dilution 1:1000), HK2 (dilution 1:2000) and GAPDH (dilution 1:10000), and subsequently the secondary antibody HRP-conjugated Affinipure Goat Anti-Rabbit IgG (H + L) (dilution 1:10000). Chemiluminescence reagents (Millipore) were used to visualize the bands of target proteins. The antibody against GAPDH was used to confirm equivalent loading. The levels of target protein bands were densitometrically determined using Image Lab Software 3.0. Representative blots are shown.

### Statistical analysis

Data are presented as means ± SD. The results were analyzed using SPSS16.0 software. The significance of difference was determined using one-way ANOVA with the post hoc Dunnett’s test. Values of *p* < 0.05 were considered to be statistically significant.

## Results

### Costunolide reduces HSC activation

**Fig. 1:**
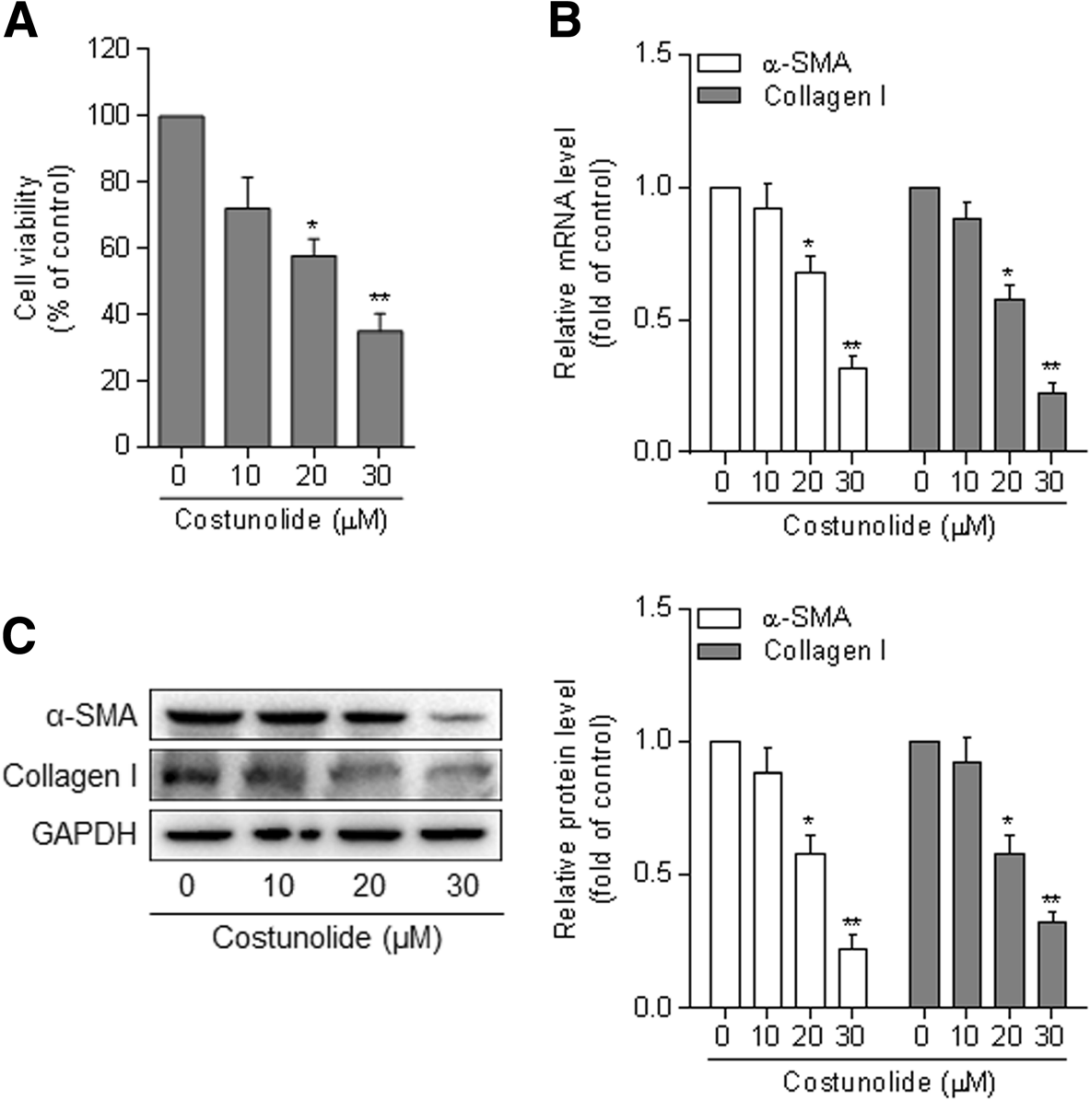
Costunolide reduces HSC activation. Primary rat HSCs at passages 3 through 5 were treated with costunolide at the indicated concentrations for 24 h. **a** – MTT assay for evaluating cell viability, which is presented as a percentage of the control. **b** – Real-time PCR for determining the mRNA expression of α-SMA and collagen I. **c** – Western blot assay for determining the protein expression of α-SMA and collagen I with quantification of the blots. Statistics: *p < 0.05, **p < 0.01 vs. control

Increased proliferation is a hallmark of HSC activation. Here, we observed that costunolide reduced the viability of HSCs in a concentration-dependent manner, and that costunolide at 20 μM caused a significant effect (Fig. 1a). Activated HSCs express α-SMA as a biomarker and produce massive type I collagen in the fibrotic liver. The mRNA levels of α-SMA and collagen I were reduced by costunolide in a concentration-dependent manner in HSCs (Fig. 1b). The protein abundance of α-SMA and collagen I was also consistently decreased by costunolide in HSCs (Fig. 1c). These results demonstrate that costunolide reduces HSC activation.

### Blocking aerobic glycolysis contributes to costunolide reduction of HSC activation

**Fig. 2:**
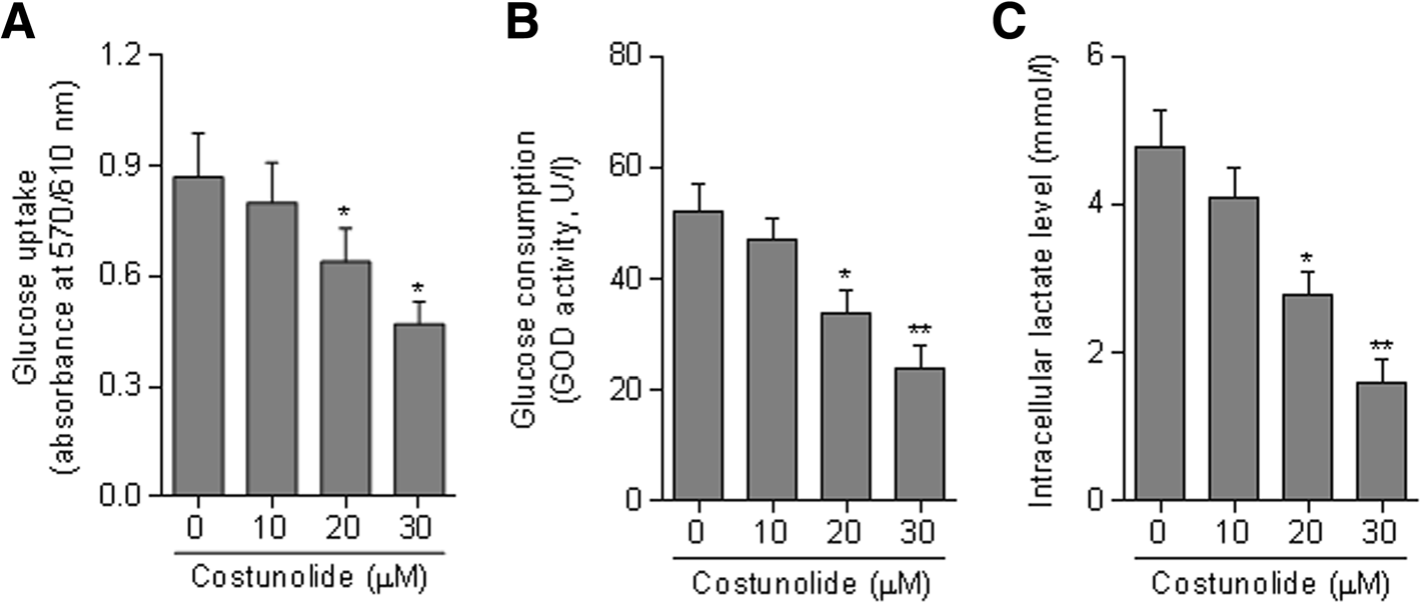
Costunolide blocks aerobic glycolysis in HSCs. Primary rat HSCs at passages 3 through 5 were treated with costunolide at the indicated concentrations for 24 h. **a** – Measurement of glucose uptake using absorbance at 570/610 nm. **b** – Measurement of glucose consumption represented by GOD activity. **c** – Measurement of intracellular lactate levels. Statistics: *p < 0.05, **p < 0.01 vs. control

We next examined the effects of costunolide on aerobic glycolysis in HSCs. The data show decreases in glucose uptake and consumption in HSCs treated with costunolide (Fig. 2a and b). The intracellular levels of lactate, the end-product of glycolysis, were reduced by costunolide in a concentration-dependent manner (Fig. 2c). These findings indicate that the glycolytic flux is efficiently blocked by costunolide.

**Fig. 3:**
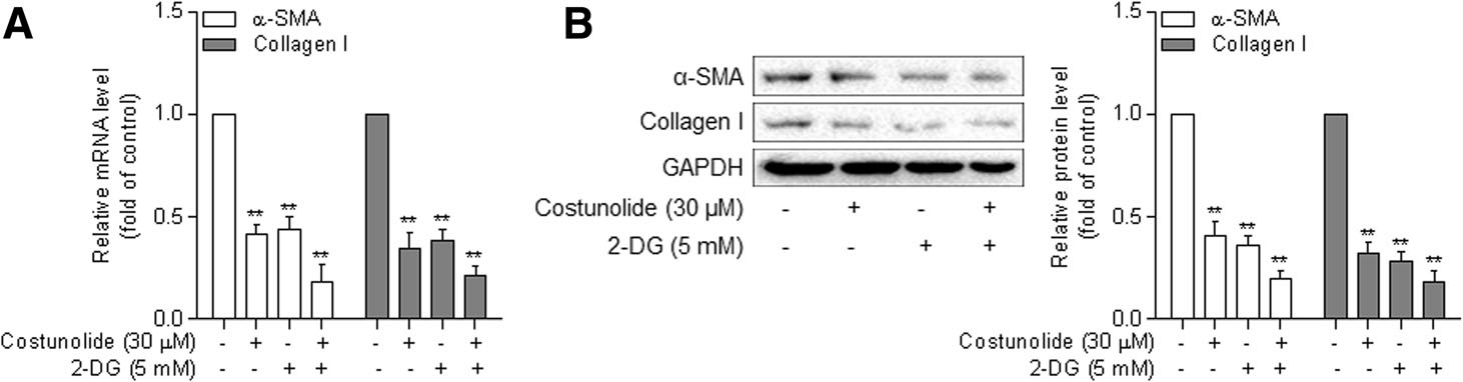
Blockade of aerobic glycolysis contributes to the reduction in HSC activation by costunolide. Primary rat HSCs at passages 3 through 5 were treated with costunolide and/or 2-DG at the indicated concentrations for 24 h. **a** – Real-time PCR for determining the mRNA expression of α-SMA and collagen I. **b** – Western blot assay for determining the protein expression of α-SMA and collagen I with quantification of the blots. Statistics: **p < 0.01 vs. control

To establish the link between blocked glycolysis and reduced viability, we used the glycolysis inhibitor 2-DG as a tool compound, and found that at 10 mM, similarly to costunolide at 30 μM, 2-DG significantly downregulated the mRNA expression of α-SMA and collagen I in HSCs (Fig. 3a). The combination of the two compounds produced more significant effects. Consistent alterations were recaptured at the protein level in HSCs treated with costunolide and/or 2-DG (Fig. 3b). These observations suggest that blocking aerobic glycolysis is critically involved in costunolide reduction of HSC activation.

### Suppression of HK2 is required for costunolide to reduce glycolysis-associated HSC activation

**Fig. 4:**
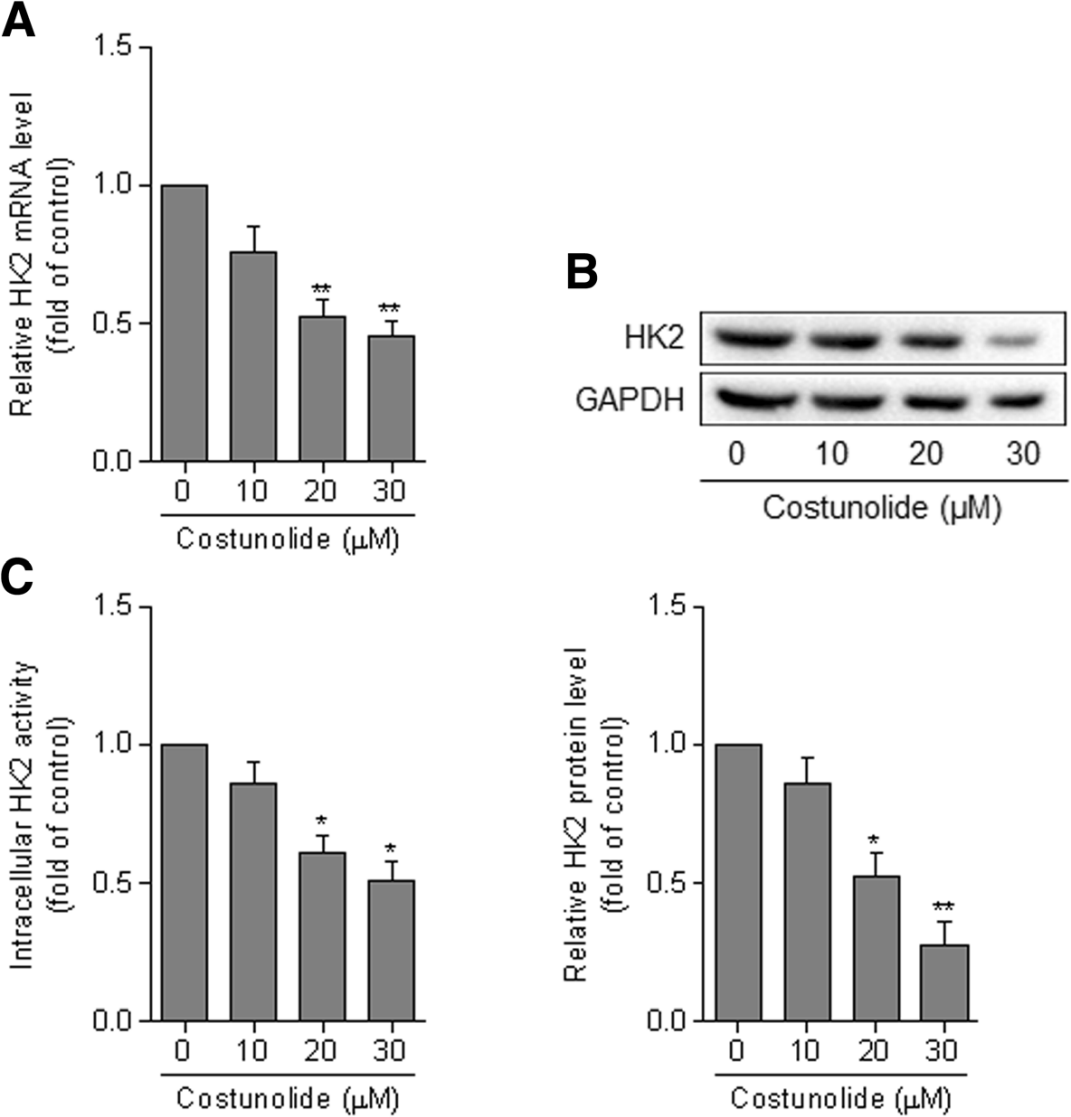
Costunolide suppresses the expression and activity of HK2 in HSCs. Primary rat HSCs at passages 3 through 5 were treated with costunolide at the indicated concentrations for 24 h. **a** – Real-time PCR for determining the mRNA expression of HK2. **b** – Western blot assay for determining the protein expression of HK2 with quantification of the blots. **c** – Measurement of the intracellular activity of HK2. Statistics: *p < 0.05, **p < 0.01 vs. control

**Fig. 5:**
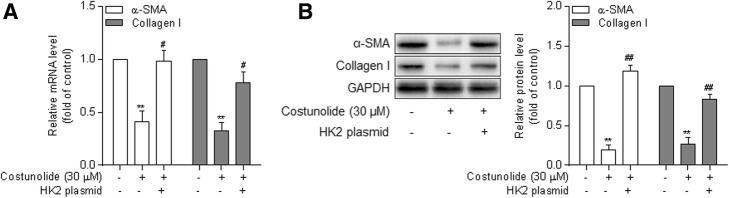
Suppression of HK2 is required for costunolide inhibition of HSC. Primary rat HSCs at passages 3 through 5 were treated with costunolide at the indicated concentrations or transfected with HK2 overexpression plasmid for 24 h. **a** – Real-time PCR for determining the mRNA expression of α-SMA and collagen I. **b** – Western blot assay for determining the protein expression of α-SMA and collagen I with quantification of the blots. Statistics: **p < 0.01 vs. control; ^#^*p* < 0.05, ^##^*p* < 0.01 vs. costunolide

We subsequently investigated the potential key molecule mediating the effects of costunolide on HSC activation. It has been acknowledged that HK2 is a pivotal rate-limiting enzyme controlling the glycolytic flux. Here, we observed that costunolide downregulated the mRNA and protein expression of HK2 in a concentration-dependent manner in HSCs (Fig. 4a and b). Moreover, the intracellular activity of HK2 was also decreased by costunolide (Fig. 4c). Interestingly, we further found that overexpression of HK2 significantly rescued costunolide-mediated downregulation of α-SMA and collagen I at both the mRNA and protein levels in HSCs (Fig. 5a and b). These results suggest that suppression of HK2 is required for costunolide to inhibit glycolysis-associated HSC activation.

## Discussion

Natural products are a potential source of novel hepato-protective drugs. The roots of *Vladimiria souliei* Ling, a medicinal herb that is widely distributed in China, have been used to improve abdominal pain, vomiting, borborygmus and diarrhea for centuries in the system of traditional Chinese medicine. Recent phytochemical studies identified the sesquiterpene lactones as the major active components that can be isolated from this plant [17].

Costunolide is a well-characterized sesquiterpene lactone compound [17]. Several pharmacological investigations have shown the potential therapeutic benefits of costunolide for liver diseases. For example, costunolide can significantly attenuate pathological changes in the liver in mice [12]. It also reduced serum levels of inflammatory factors in lipopolysaccharide- and d-galactosamine-induced acute liver injury in mice. These effects were associated with its suppression of NF-κB activation [12]. Further studies demonstrated that its protective mechanisms could be linked to the enhanced anti-oxidative defense system and prevention of hepatocyte apoptosis [13].

Our current study is the first to evaluate the effects of costunolide on HSC activation implicated in the treatment of hepatic fibrosis. We isolated primary HSCs from rats for the experiments. The freshly isolated HSCs are spontaneously activated in culture, faithfully mimicking the activation process during hepatic fibrogenesis in vivo. Thus, they are the ideal cell model for studying the biological properties of HSCs and pharmacological intervention [14].

We found that costunolide reduced the viability and decreased the expression of α-SMA, a well-established marker of activated HSCs. Importantly, the expression of collagen I, the major component of the ECM during hepatic fibrogenesis, was downregulated by costunolide in the primary HSCs. These results strongly indicate that costunolide suppresses the pro-fibrogenic properties of activated HSCs.

We next explored the potential mechanism underlying costunolide suppression of HSC activation. Increasing evidence supports the notion that pharmacologically blocking aerobic glycolysis could be a novel strategy for reducing HSC activation and attenuating liver fibrosis [3]. For example, curcumin was found to inhibit the expression of several key molecules involved in glycolysis, leading to decreased viability and increased apoptosis in HSCs [18]. Activation of AMPK was required for curcumin blockade of HSC glycolysis [19]. All three rate-limiting enzymes of glycolysis (HK2, PFK1 and PKM2) were inhibited by oroxylin A in HSCs, resulting in the restriction of HSC contraction [20].

Our results here suggest that costunolide is also a natural compound that blocks aerobic glycolysis. It reduced glucose uptake and consumption, and decreased lactate production. The glycolysis inhibitor 2-DG had similar reducing effects on the expression of HSC activation markers.

These discoveries suggest an association between blocking glycolysis and inhibiting HSC activation. This strengthens the possibility of developing natural products that target the glycolytic pathway to halt HSC activation. It could be assumed that interruption of glucose glycolysis reduces the amount of metabolic intermediates that are indispensable for the synthesis of amino acids, nucleotides and lipids, leading to the suppression of HSC activation. To the best of our knowledge, this is the first report describing the effects of costunolide on cellular glucose metabolism with therapeutic implications.

We further investigated the linking molecule involved in costunolide blocking glycolysis and suppression of HSC activation. We mainly examined the effects of costunolide on the rate-limiting enzyme HK2, and indeed observed that the de novo synthesis and intracellular activity of HK2 was inhibited by costunolide in HSCs. HK2 plays a pivotal role in glycolysis and cell metabolism. There is evidence that HK2 was overexpressed in activated HSCs during hepatic fibrogenesis [4]. HK2 can support the highly glycolytic phenotype after rapid entry of glucose into HSCs on the glucose transporter. HK2 can bind to both ATP and incoming glucose, producing the product glucose-6-phosphate at an elevated rate. This key metabolite then functions both as a biosynthetic precursor to support cell growth and as a precursor for lactate, causing an unfavorable environment for hepatic cells [21].

Here, we further observed that overexpression of HK2 significantly abolished the inhibitory effects of costunolide on the expression of HSC activation markers. These data indicate that costunolide repression of HSC activation is dependent on inhibition of HK2. However, we could not conclude that HK2 was a direct target molecule for costunolide effects. Sequence analysis of the HK2 promoter revealed well-defined cis-elements for transcription initiation, and cis-elements for activation by PKA and PKC/RAS pathways [22–24]. Whether these transcription mechanisms were involved in costunolide inhibition of HK2 in HSCs awaits further investigation.

Our current discoveries demonstrate that costunolide reduces the viability and activation of HSCs, and that this is associated with the blocking of aerobic glycolysis via inhibition of HK2. We confirmed the strategy of reducing HSC activation through interference with aerobic glycolysis, and suggest costunolide as a promising antifibrotic candidate for further development.

## Data Availability

Please contact the author with data requests.

## Abbreviations

2-DG: 2-Deoxy-D-glucose
ECM: Extracellular matrix
GAPDH: Glyceraldehyde phosphate dehydrogenase
GOD: Glucose oxidase
α-SMA: α-Smooth muscle actin
HSC: Hepatic stellate cells
HK2: Hexokinase 2
MTT: 3-(4,5-Dimethylthiazol-2-yl)-2,5-diphenyl tetrazolium bromide
